# Bilateral C1–C2 Transarticular Screw and C1 Laminar Hook Fixation and Bone Graft Fusion for Reducible Atlantoaxial Dislocation: A Seven-Year Analysis of Outcome

**DOI:** 10.1371/journal.pone.0087676

**Published:** 2014-01-31

**Authors:** Xiang Guo, Bin Ni, Ning Xie, Xuhua Lu, Qunfeng Guo, Ming Lu

**Affiliations:** Department of Orthopedics, Changzheng Hospital, The Second Military Medical University, Shanghai, P.R. China; Toronto Western Hospital, Canada

## Abstract

**Background:**

Bilateral C1-2 transarticular screw and C1 laminar hook fixation was developed on the basis of transarticular screws fixation. The modified technique has showed a better biomechanical stability than established techniques in previous study. However, long-term (minimum follow-up 7 years) outcomes of patients with reducible atlantoaxial dislocation who underwent this modified fixation technique have not still been reported.

**Methods:**

A retrospective study was conducted to evaluate the outcome of 36 patients who underwent this modified technique. Myelopathy was assessed using the Ranawat myelopathy score and Myelopathy Disability Index. Pain scores were assessed using Visual Analogue Scale. Radiological imaging was assessed and the following data were extracted: the atlantodental intervals, the space available for cord, presence of spinal cord signal change on T2 weighted image, C1–C2 angle, C2–C7 angle and fusion rates.

**Findings:**

All patients achieved a minimum seven-year follow up. 95% patients with neck and suboccipital pain improved after surgery; in their Visual Analogue pain scores, there was a greater than 50% improvement in their VAS scores with a drop of 5 points on the VAS (P<0.05). 92% of patients improved in the Ranawat myelopathy grade; the Myelopathy Disability Index assessment showed a preoperative mean score of 35.62 with postoperative mean 12.75(P<0.05). There was not any significant atlantoaxial instability at each follow-up time. The space available for cord increased in all patients. Postoperative sagittal kyphosis of the subaxial spine was not observed. After six months after surgery, bone grafts of all patients were fused. No complications related to surgery were found in the period of follow-up.

**Conclusions:**

The long-term outcomes of this case series demonstrate that under the condition of thorough preoperative preparations, bilateral C1–C2 transarticular screw and C1 laminar hook fixation and bone graft fusion is a reliable posterior atlantoaxial fusion technique for reducible atlantoaxial dislocation.

## Introduction

Atlantoaxial dislocation caused by fractures, rheumatoid arthritis, congenital deformities or traumatic lesions of the transverse ligament [1], often results in acute or chronic spinal cord compression, a possible threat to a patient’s life if immediate reduction has not been achieved. Even though conservative management, including halo brace or cast, could be appropriate for few patients, such as atlantoaxial rotatory subluxation or avulsing fracture of the tubercle for insertion of the transverse ligament, surgical intervention is usually necessary for most patients who suffer from atlantoaxial dislocations. The goal of surgical intervention is to reconstruct the stability of atlantoaxial articulation.

Conventional posterior atlantoaxial fixation techniques, such as Gallie wiring and C1-2 transarticular screw, although considered to be successful for a long time, are frequently associated with high rates of pseudoarthrosis and internal fixation breakage [2], [3]. From a biomechanical point of view, fixation techniques of the atlantoaxial articulation can be divided into three different types. One-point fixation stabilizes the motion segment merely from posterior with a structural bone graft (e.g. Gallie wiring, Halifax clamps). Two-point fixation stabilizes the articulation with C1-2 transarticular screws laterally placed into C1-2 lateral mass or bilateral C1 lateral mass screws combined with C2 isthmic screws. Three-point fixation consists of the combination of the two previous types, such as transarticular screw combined with Gallie wiring, thus stabilizing the C1–C2 motion segment both laterally and posteriorly. In previous biomechanical studies it has been shown that the three-point fixation is superior to both two- and one-point fixations [4], [5].

**Figure 1:**
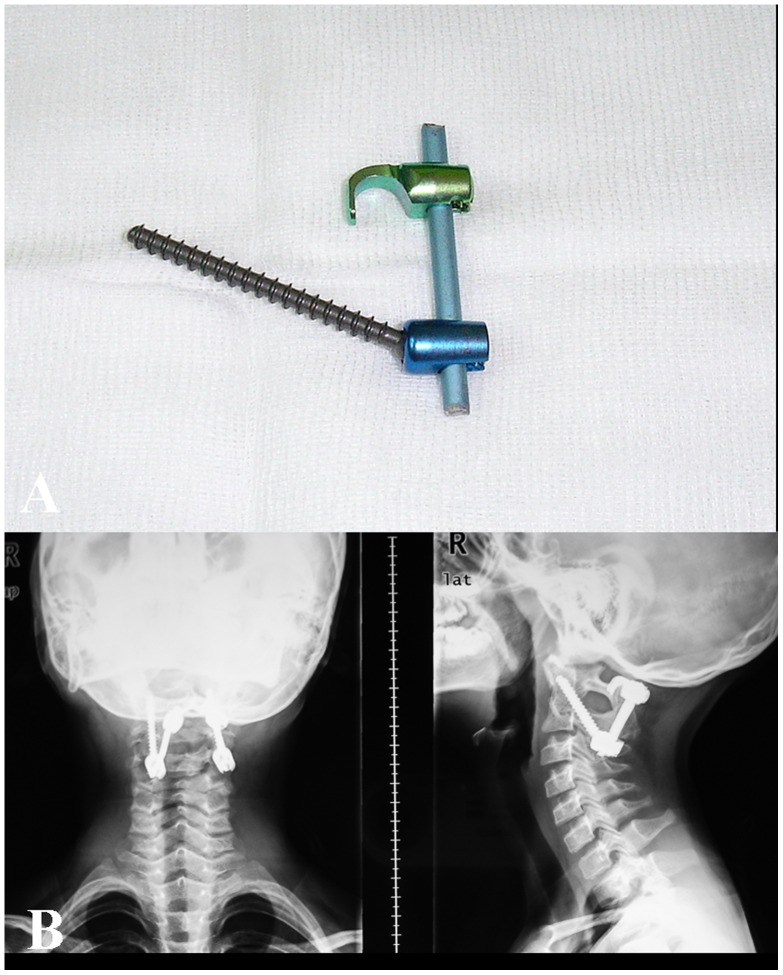
A modified implant and postoperative radiograph of patient 1. A color photograph (**A**) showing C1-2 transarticular hooks and C1 laminar hooks implant. Postoperative lateral and anteroposterior view radiographs (**B**) demonstrating the satisfactory position of implants.

In 2004,the authors [6] developed a modified posterior atlantoaxial fixation technique for the atlantoaxial instability on the basis of established posterior atlantoaxial fixation techniques. This technique uses one pair of parallel placed inner fixation systems in which C1-2 transarticular screws are combined with C1 laminar hooks (Fig. 1), and they are connected each other with a rod and connectors to construct a three-point fixation. From March, 2004 to May, 2006, this fixation technique was applied to 36 patients who suffered from reducible atlantoaxial dislocations. The purpose of this article was to report long-term (minimum follow-up 7 years) clinical and radiologic outcome of this case series of consecutive patients, who underwent this modified fixation technique.

## Materials and Methods

### Ethics Statement

Ethics Committee of Changzheng hospital of Shanghai approved this retrospective study and the signed consent was given by all patients for their information to be stored in the hospital database and used for research.

A retrospective studies were conducted to evaluate outcome of 36 patients who underwent bilateral C1-2 transarticular screws and C1 laminar hooks fixation following routine cranial traction of one week. In the case series, none of these patients had occipitoatloid or subaxial cervical spine problems. Etiology included traumatic lesions of the transverse ligament (n = 14), odontoid fractures (n = 12),Os odontoideum (n = 7), C1-2 rheumatoid arthritis (n = 3). The preoperative atlanto-dens interval mean was 6.4 mm (range, 5.4–9.6); all patients were diagnosed as atlantoaxial instability with injury of transverse ligament. All were operated on by a single surgeon at our institutions and all patients with 7 years or more follow-up were included in this study.

Clinical data, including patients symptoms, the extent of the myelopathy (if present), and the severity of the pain, were collected at both pre-operation and the final point of follow-up. The patients’ pain extent was assessed using the Visual Analogue Scale. Myelopathy was assessed using the Ranawat Myelopathy Score and the Myelopathy Disability Index. Moreover, any complications related to surgery were investigated. The patients were examined by the spinal surgery team during their outpatient follow-up.

The radiologic images, including neutral,flexion-extension lateral radiographs, MRI, thin cut CT scans with three-dimensional reconstructions, were assessed and the following data were extracted at both pre-operation and the final point of follow-up: the atlanto-dens interval (ADI), the space available for the cord (SAC), the presence of signal change in the spinal cord on T2 weighted image, the C1-2 angle that was defined as the angle subtended by a line drawn parallel to the inferior aspect of C-1 and a line drawn parallel to the inferior end plate of C-2, and the C2-7 angle was defined as the angle subtended by a line drawn parallel to the posterior border of the C-2 vertebral body and a line drawn parallel to the posterior border of the C-7 vertebral body. Moreover, the positions of C1-2 transarticular screws and the stability of atlantoaxial articulation were evaluated. Bone fusion was determined on the basis of the presence of trabeculated bone in the sagittal reconstruction images of CT and no atlantoaxial instability in flexion/extension radiographs.

The SPSS 10.0 software was used to analyze the data as mean ±standard deviation (Mean±SD). A one-way analysis of variance (ANOVA) was used to compare multiple data sets, and paired t-test was used to perform pairwise comparison. Differences were considered significant if the *P* value was smaller than 0.05.

### Preoperative Imaging Evaluation and Surgical Procedure

A thorough preoperative imaging analysis of the atlantoaxial articulation, based on three-dimensional CT scan, is conducted to evaluate whether there is a high-riding VA under the lateral mass of C2 for the safe placement of the C1-2 transarticular screw. All patients achieved a satisfactory reduction of C1-2 articulation via cranial traction for one week before surgery.

The patient is placed in the prone position while maintaining C1–C2 reduction confirmed with C-arm fluoroscopy. Posterior midline cut was made from the C1 to the C2 spinal process. The Vertex laminar elevator was used to strip away the soft tissue above and below the posterior arch of C1. The suitable situation between Vertex laminar elevator and the posterior arch of C1 was inspected. Because the Vertex laminar hook was mainly designed for anatomic structure of laminar of subaxial cervical spine, occasionally the high-speed drill was used to fix the posterior arch of C1 to fit the contour of the Vertex laminar hook. The Vertex laminar hook was placed to hang on the posterior arch of the atlas. The placement of the transarticular screw was monitored under the C-arm fluoroscopy or radiography. A hole was bored at approximately 2 mm superior-lateral the inner edge of the C2 inferior articular process. The 3.5 mm drill bit was used to drill cautiously into to the C2 isthmus. The appropriate drill direction was determined according to the C-arm fluoroscopy imaging. The drill bit followed along the sagittal surface, near the inner surface of the isthmus of the C2 and passed through the posterior part of the atlantoaxial articulations and went into the lateral mass of C1. The screw must enter along the pre-designed angle, to avoid penetrating the cortical bone and damaging the vertebral artery and the spinal cord. A small ball probe was used to palpate the length and wall of the hole to guarantee that no cortical breakthroughs into the spinal canal had occurred. The depth of the screw hole should be measured carefully in order to choose appropriate screw length. A 4.0 mm polyaxial cortical screw was cautiously inserted along the same trajectory. The same procedure was used to implant the opposite side screw. Next, the rod was cut into the appropriate size and pre-curved, then fitted into the top open of the C1 laminar hook and the top open of the polyaxial transarticular screw. At last, nuts were twisted in order to achieve temporary Fixation.

A bone block, approximately 2×1×1 cm, was harvested from the right posterior iliac crest in the usual manner. The bone transplantation bed was prepared. The high-speed drill was used to remove the cortical bone at the lower part of the C1 posterior arch, as well as at the upper edge of the C2 laminar, and a superior notch was made in the spinous process of C2. The bone block was inserted between the C1 posterior arch and the C2 spinous process and wedged between the C1 and C2 connection rods. Compressor was used to compress the inner fixation systems at both sides at the same time in order to lock the bone block between the C1 posterior arch and the C2 spinous process. Finally, the nuts were completely tightened. With this implant, the posterior arch, the autogenous bone block, and the same side C2 laminar were fixed together.

Surgery time ranges from 70 to 90 minutes. Blood loss of atlantoaxial fixation using bilateral C1-2 transarticular screw and C1 laminar hook is about 400 ml. All patients need Philadelphia collar support for three months.

## Results

### Clinical Outcomes

**Figure 2:**
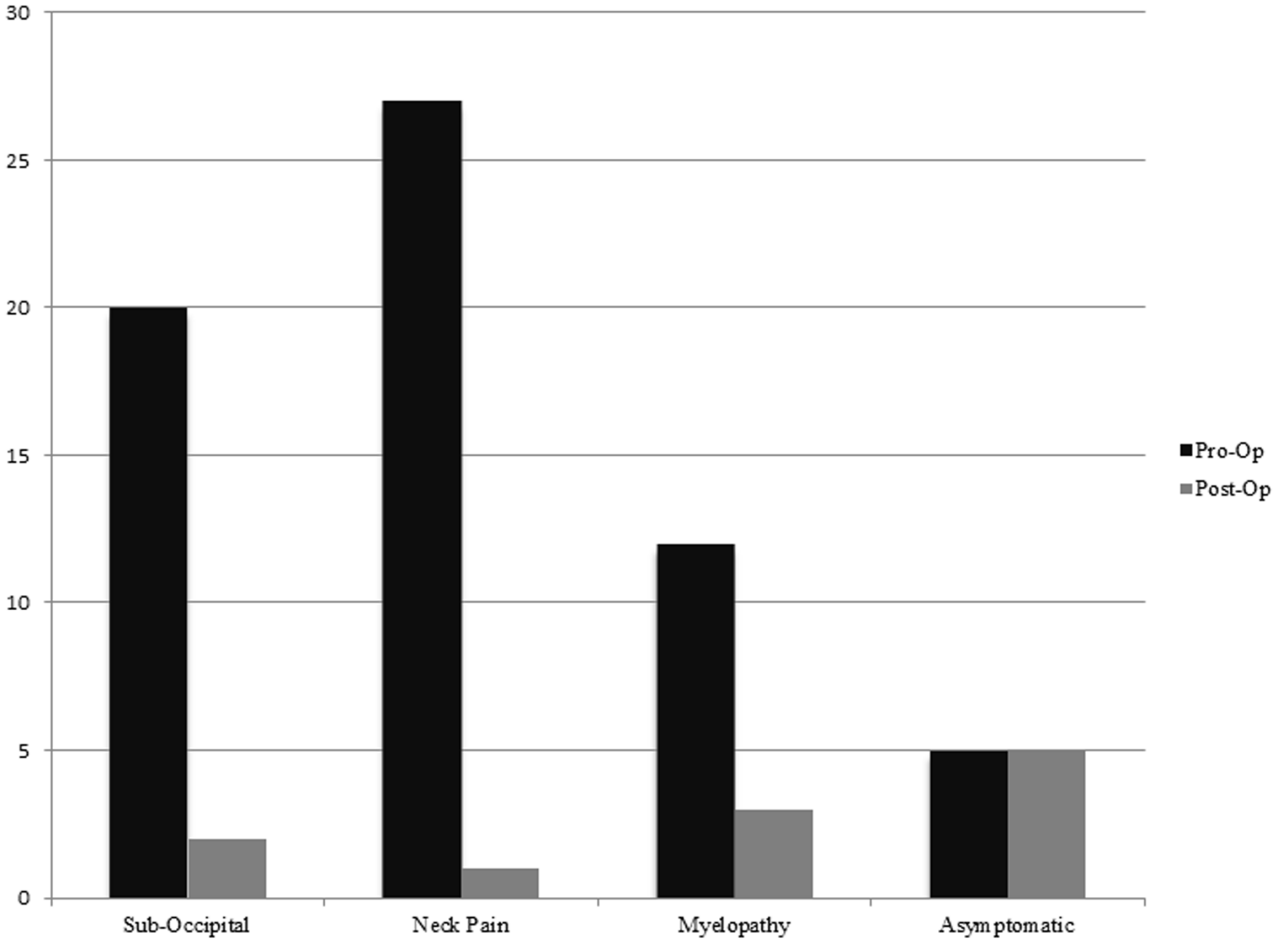
Presenting symptoms before surgery compared to after surgery.

All patients achieved a minimum seven year follow up. There were 7 males and 29 females. The median age was 43 (range 24–65) years. The average duration of neck symptoms was 3.6 (range 0.5–48) months. Before surgery,patients were classified according to the following symptoms: suboccipital pain in 20, neck pain in 27, myelopathy in 12 and asymptomatic 5. After surgery, the number of patients who had complaints decreased: suboccipital pain, 2; neck pain, 1; and myelopathy, 3 (Table 1, Figure 2). 95% percent patients with neck and suboccipital pain improved after surgery in their Visual Analogue pain scores, who have a greater than 50% improvement in their VAS scores with a drop of 5 points on the VAS. There was a statistically significant difference between their pre and postoperative pain scores (P<0.05).

**Table 1:** Presenting Symptoms Preoperatively Compared to Postoperatively (n = 36).

	SubOccipital	NeckPain	Myelopathy	Asymptomatic
Preoperatively	20	27	12	5
Postoperatively	2	1	3	5

**Figure 3:**
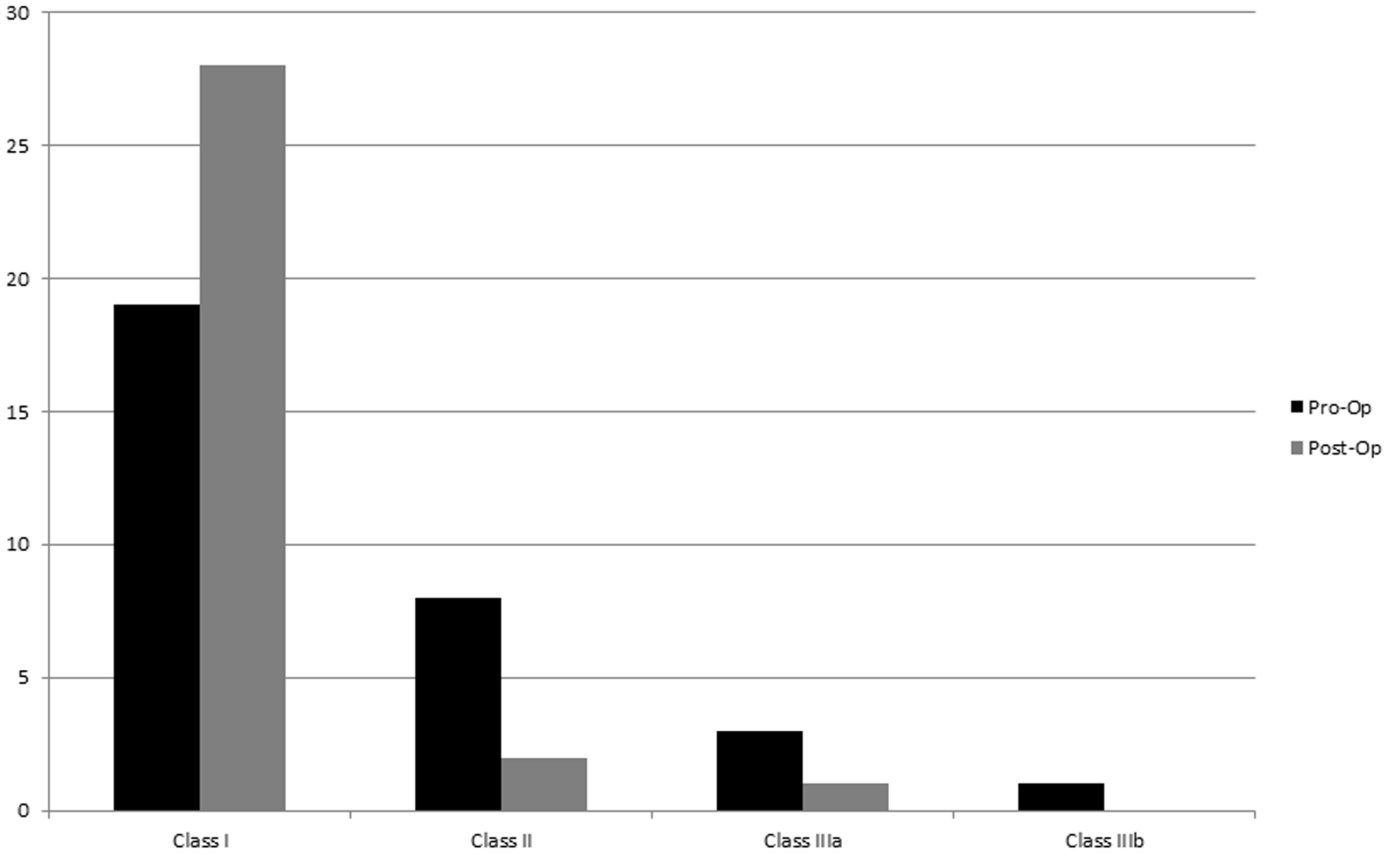
Ranawat myelopathy score before surgery compared to after surgery.

The preoperative Ranawat myelopathy grade [7] was respectively class I in 19, class II in 8, and class IIIa in 3, class IIIb in 1. After surgery, there was an improvement in the majority of the patient’s myelopathy grade: 28 patients were in class I (7 patients from class II and 2 patients from class IIIa); 2 patients were in class II (1 patient from class IIIa and 1 patient unchanged); 1 patient was in class IIIa, (1 patient from class IIIb). (Table 2, Figure 3). The Myelopathy Disability Index assessment [8] showed a preoperative mean score of 35.62 with postoperative mean 12.75. (P<0.05, paired *t-* test). Any fixation failure and neurovascular injury related with these operations did not occur. There was no perioperative mortality in this series. At the time of follow-up, 2 patients was found to have died but their deaths were unrelated to their surgery and occurred more than 6 months after surgery.

**Table 2:** Ranawat Myelopathy Score Preoperatively Compared to Postoperatively (n = 36).

	Class I(Normal)	Class II	Class IIIa	Class IIIb
Preoperatively	19	8	3	1
Postoperatively	28	2	1	0

### Radiological Outcomes

The atlanto-dens interval is the distance between the dorsal edge of the anterior arch of the atlas and the ventral edge of the odontoid process. The normal ADI is less than 3 mm in adults and less than 4 mm in children. [9], [10] An ADI between 3 to 5 mm in adults indicates a disruption of the transverse ligament, ADI between 5 to 10 mm represents additional ligamentous damage and an ADI of greater than 10 mm indicates complete ligamentous disruption. [11] The SAC is the distance from the ventral edge of the posterior ring of atlas or foramen magnum to the dorsal aspect of the odontoid process. Spinal cord compression always occurred when the SAC was 14 mm or less and never occurred if the SAC was 18 mm or more. [12].

The atlanto-dens interval improved in all patients–preoperative mean was 6.4 mm (range, 5.4–9.6) as compared to averaged 2.34 mm postoperatively. There was no unstable appearance in the atlantoaxial joint on the lateral flexion-extension radiograph at each follow-up point. The space available for the cord increased in all patients–preoperative mean was 13.54 mm (13–24) as compared to averaged 19.93 mm in the postoperative period. 10% patients still had signal change within the cord on MRI T2WI six months after surgery. The mean C1–2 angles was 21.3±6.3° before surgery, and 26.4±4.6° after surgery; there were on patients who have a atlantoaxial hyperlordosis(at >30°) following C1-2 fixation. The mean C2–7 angles were 19.4±5.8° before surgery and 15.8±7.8° after surgery; Postoperative sagittal kyphosis of the subaxial spine was not seen in the case group.

Moreover, of 72 C1-2 transarticular screws related to 36 patients, 3 (4.2%) screws cut out dorsally in the isthmus of C2, which are related to 2(5.5%) patients. For the rest 69(95.8%) screws, satisfactory positions were achieved. At 3 months after surgery, 35 patients(97.2%)achieved complete fusion of the bone graft besides 1 patients (2.7%) who suffered from osteoporosis, and after be reinforced in the treatment of osteoporosis and continually fixed with collar for three months, the bone graft was fused.

## Discussion

Treatment of reducible atlantoaxial instability can be challenging. The goal of surgical intervention is to reconstruct the stability of atlantoaxial articulation. There are a number of fixation techniques including posterior wiring, Apofix clamp, Goel-Harms constructs, C1–2 transarticular screws and C1-2 transarticular screw combined with Gallie technique. Each type of technique has a unique set of risks and benefits and the choice of technique is related to the anatomy of the patient and their disease process and to the familiarity of the surgeon with any particular technique.

Wiring techniques, such as Gallie and Brooks technique, are still used currently. However, they are considered less biomechanically stable compared to C1-2 transarticular screw and Goel-Harms techniques.[4], [5], [13]–[16] Also, they carry a neurologic risk because sublaminar wires are passed under the C1 arch and in order to achieve good bone graft fusion, the patient operated on the Gallie or Brooks technique must be placed in a solid external fixation after surgery, such as a halo brace, which usually results in some related complications, for example skin infection and leakage of cerebrospinal fluid.

Apofix laminar clamp uses laminar hook to fix the C1-2 posterior structure, thus avoids the neurologic risk caused by sublaminar wires that are passed under the C1 arch, however it is still a simple one-point fixation, and its biomechanical stability do not notably improve.

Goel-Harms technique composed of C1 lateral mass screws coupled to C2 pedicle screws has been reported to be an effective method to provide a solid fixation construct in patients with atlantoaxial instability. [17] However, because of abundant venous plexus around insert point, the placement of C1 lateral mass screw becomes difficult. Additionally, from a theoretical biomechanical viewpoint, it is still a one-point fixation, but as the screws are rigidly fixed to the longitudinal members of the construct, its biomechanical properties are probably superior to Gallie wiring and alone C1-2 transarticular technique. [15], [18].

C1-2 transarticular screw has been reported to contribute a solid stability in patients with atlantoaxial instability.[19]–[22] However, there were still some reports that the C1-2 transarticular screw was broken and a bone graft block was needed for nonunion after surgery. [23] From a theoretical biomechanical viewpoint, it is merely a two-point fixation and cannot provide the good stability in 3-D motion of the atlantoaxial articulation, especially in extension and flexion [5]. Naderi et al [24] pointed out that increasing the number of fixation points in atlantoaxial fixation could further limit the C1-2 motion.

Therefore, to further limit the motion of atlantoaxial articulation and to achieve the long-term solid stability, the modified techniques that mutually compromise the C1-2 transarticular screw technique are required [5], [24]. Bilateral C1-2 transarticular screws and C1 laminar hooks fixation not only fully inherits the essence of a “three-point” fixation, but also significantly enhances the ability to limit anterior-posterior motion. As a matter of fact, it has been proved by authors’ previous biomechanical study [6]that bilateral C1-2 transarticular screws and C1 laminar hooks fixation can result in better biomechanical fixation construct compared with established C1-2 fixation techniques. Furthermore, in the seven-year retrospective research, the results have showed that this modified posterior fixation technique has an excellent clinical outcome. As a modified C1-2 fixation technique, the indications include all conditions suitable for C1-2 transarticular screw, such as reducible atlantoaxial instability and chronic type II of odontoid fracture; on the contrary, the contraindications include congenital absence or fracture of posterior arch of C1, neurovascular deformity of atlantoaxial complex and irreducible atlantoaxial dislocation.

Olerud Cervical Fixation to manage odontoid fractures and C1–C2 instability in rheumatoid arthritis was introducte by Cornefjord et al [18] in 2003. This fixation system composed of bilateral transarticular screws and atlas claws is basely similar to the authors’ fixation technique except for C1 hooks used in authors’ fixation technique. Although atlas claws would be more rigid against extension motion of C1-2 than hooks because simultaneously finish a fixation at the superior and inferior edge of posterior arch of C1, a bone block, as a bone graft in the authors’ technique, resulted into a more rigid construct as a whole than bone chips or strips that can restrict the extension motion strongly, as descried in previous study, [15], [25] which makes up for the drawback that the atlas hooks can merely restrict extension motion of C1-2 rigidly. Moreover, compressor was used to compress the inner fixation systems at both sides at the same time in order to lock the bone block between the C1 posterior arch and the C2 spinous process., which maintains the stability of the bone graft and improves the fusion rate because compression of a bone graft is considered to be a factor necessary for fusion to occur [26].

It may be argued that there are risks of vertebral artery injuries in all methods using a C1-2 transarticular screw. [3], [27] Although some anatomic or radiologic studies of the atlantoaxial region have considered that in up 20% of the cases, the proper placement of the C1-2 transarticular screw is not possible mainly because of a high-riding vertebral artery (VA), [3], [20], [28], [29] the accuracy of placing C1-2 transarticular screws is approximately 96% in this case series. Certainly, the higher accuracy to place C1-2 transarticular screw must base on a sufficient preoperative preparation and skillful surgical technique. Firstly, preoperatively a satisfactory reduction of atlantoaxial articulation is required, which not only significantly ameliorates the compression of spinal cord caused by a posterior displacement of odontoid process of C2, but also creates the beneficial condition for the safe placement of C1-2 transarticular screw. Secondly, thorough preoperative imaging and analysis of the atlantoaxial articulations are required to evaluate whether there is a high-riding VA under the lateral mass of C2 which can cause the placement of the C1-2 transarticular screw to become greatly risky. Some authors [30] defined the high-riding VA on the basis of CT reconstruction: an internal height less than 2 mm, an isthmus height and width less than 5 mm, or both. However, the isthmus height and width is more important because the screw goes through the isthmus itself regardless of the internal height. According to our clinical experience, as patients’ C2 isthmic height and width is less than 5 mm in preoperative imaging, it was very difficult to place the C1-2 transarticular screw with diameter of 4.0 mm. Finally, the method to place screws that the insert point of the screw should be positioned at approximately 2 mm superior-outside the medial edge of the C2 inferior articular process and the screw trajectory should be steeper than usual should be used to guarantee the surgical safety, which complies with the essential principle for approaching a high-riding VA or even a normal VA that the screw should pass superiorly or superomedially to the VA groove. [31] In the cases, all patients’ C1-2 transarticular screws, including 4 screws of the 2 patients with bilateral high-riding VAs, were placed with the above insert point and screw trajectory; fortunately, there were no visible injury of the vertebral artery and the spinal cord during the operative term, and there were no delayed neurovascular injury and significant instability of the atlantoaxial articulation during the clinical follow-up term.

Regardless of surgical procedure, the major objectives of atlantoaxial fixation are reduction of atlantoaxial dislocation, reconstruction of stability of atlantoaxial articulation. However, some studied [32], [33]proposed that atlantoaxial fixation in a hyperlordotic position may cause the postoperative kyphotic sagittal alignment of the subaxial spine, and emphasized that the atlantoaxial angle should be fixed in an optimal position to maintain the physiological cervical alignment postoperatively; furthermore, some author [34], [35] suggested that the optimal angle of atlantoaxial fixation should be between 25° and 30°. In this case group, the mean C1–2 angles were 26.4±4.6° after surgery, which pertained to the range of the optimal angles; additionally, this fixation technique used a structural bone graft to finish atlantoaxial posterior fusion, which effectively prevent the pressure-induced fixation of the atlantoaxial joint in a hyperlordotic position, as previously described by Matsumoto et al. [36]; therefore, there was no case that has a kyphotic sagittal alignment of the subaxial cervical spine in the period of minimum follow-up of 7 years.

There is no consensus on the type of external orthosis following transarticular screw fixation. Several authors [22] have recommended the routine use of halo vest immobilization for a period of 6 to 12 weeks. However, other authors [37]proposed that with the C1-2 transarticular technique, a 3-point fixation is achieved and a Philadelphia collar supporting for 6 to 12 weeks is sufficient to enhance fusion. The radiologic fusion rates were 97%. In our cases, it is outstanding that bone graft fusion rate is 100% without relying on a solid external fixation. The main reason of this outstanding outcome is that this modified technique has an excellent biomechanical stability and maintains the stability of the bone graft and promote the fusion via longitudinally compressing bone graft, which is considered to be some factor necessary for fusion to occur [26].

## Conclusion

The long-term outcome of this case series of consecutive patients is promising. No postoperative neurological deficits or atlantoaxial instability and no serious complications related to the use of the device were encountered in the case series; a solid fixation and high fusion rate of bone graft without secondary kyphotic sagittal alignment of subaxial spine was achieved, which does not depend on the structural bone graft and a solid external fixation. To sum up, under the condition of thorough preoperative imaging and analysis of the atlantoaxial articulation and satisfactory reduction of the atlantoaxial articulation, bilateral C1–C2 transarticular screw and C1 laminar hook fixation and bone graft fusion is a reliable posterior atlantoaxial fusion technique. In the future, studies focusing on the multicenter prospective cohort studies may help to further validate the efficiency of bilateral C1–C2 transarticular screw and C1 laminar hook fixation and bone graft fusion.
